# Historical and Current Concepts of Fibrillogenesis and *In vivo* Amyloidogenesis: Implications of Amyloid Tissue Targeting

**DOI:** 10.3389/fmolb.2016.00017

**Published:** 2016-05-09

**Authors:** Robert Kisilevsky, Sara Raimondi, Vittorio Bellotti

**Affiliations:** ^1^Department of Pathology and Molecular Medicine, Queen's UniversityKingston, ON, Canada; ^2^Unit of Biochemistry, Department of Molecular Medicine, University of PaviaPavia, Italy; ^3^Wolfson Drug Discovery Unit, Division of Medicine, Centre for Amyloidosis and Acute Phase Proteins, University College LondonLondon, UK

**Keywords:** historical perspectives, *in-vivo/in-vitro* comparisons, site specific amyloid tissue deposition

## Abstract

Historical and current concepts of *in vitro* fibrillogenesis are considered in the light of disorders in which amyloid is deposited at anatomic sites remote from the site of synthesis of the corresponding precursor protein. These clinical conditions set constraints on the interpretation of information derived from *in vitro* fibrillogenesis studies. They suggest that in addition to kinetic and thermodynamic factors identified *in vitro*, fibrillogenesis *in vivo* is determined by site specific factors most of which have yet to be identified.

## Introduction

**Figure 1:**
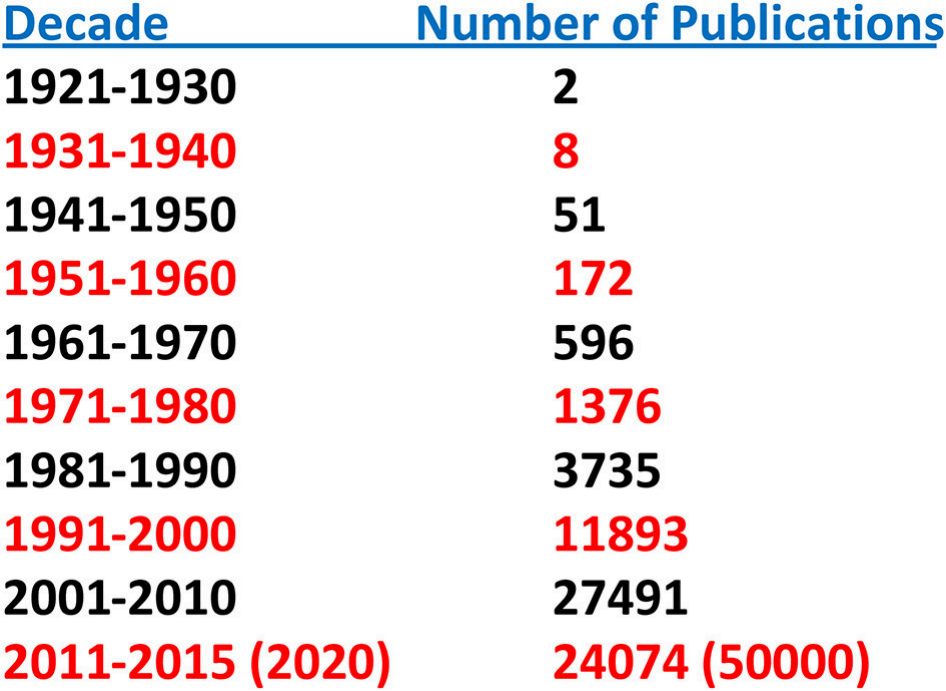
**“Amyloid” publications by decade determined from pubmed**.

**Figure 2:**
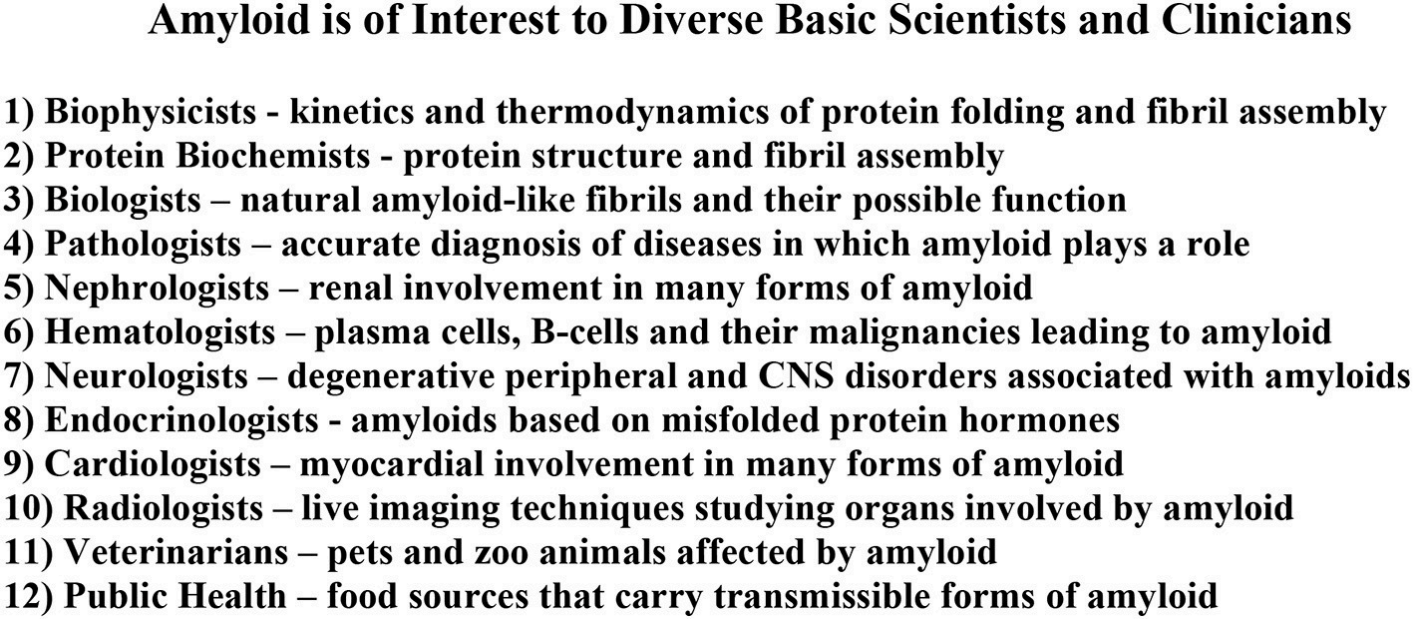
**The broad diversity of interests in amyloid**.

Until relatively recently “amyloid” was considered to be a relatively rare and esoteric medical entity. A “Pubmed” search of the term “amyloid” by decade indicates but two publications for the period 1921–1930 and eight for 1931–1940. This increase in number accelerated over the next four decades and reached ~25,000 for the period 2001–2010. Given the number of publications for 2011–2015 the projected result for 2011–2020 is of the order of 50,000 (Figure 1). Furthermore, amyloid involvement in common disorders such as Alzheimer's disease and type 2 diabetes as well as forms (e.g., prions) that may be transmitted through our food supply have made it a subject of interest to diverse clinicians and basic scientists (Figure 2). Most importantly existing therapeutic modalities that prevent continuous amyloid deposition allows the mobilization of existing deposits with clinical improvement. Such observations indicate that amyloid does turn over and its presence in tissue has adverse effects on physiological function. Amyloid is not simply a “tombstone” of previous injuries.

The history of “amyloid” as it relates to its composition, structure, and the pathogenetic mechanism of tissue deposition has been the subject of several extensive recent reviews (Sipe and Cohen, 2000; Kyle, 2001; Westermark, 2005). We will focus primarily on more modern concepts that may promote, or potentially confound, future progress. This is particularly true of *in vivo* amyloidogenesis that occurs at tissue sites remote from the biosynthetic origin of the corresponding amyloid precursor protein. For example, in animal models that mimic the form of human amyloid seen after persistent acute inflammation, amyloid is derived from the acute-phase protein serum amyloid A (SAA). This protein is synthesized primarily in the liver but is first deposited in very specific anatomic sites such as the follicular and perifollicular zones of the spleen, which are remote from its site of synthesis (Snow and Kisilevsky, 1985). More remarkably a different amyloidogenic protein, transthyretin (TTR), apparently has different tissue affinities after the substitution of but single amino acids, products of genetic mutations (Benson, 1996; Benson and Uemichi, 1996; Saraiva, 2001). A similar observation has been made recently in the case of beta-2-microglobulin (β2M; Valleix et al., 2012; Mangione et al., 2013). Why and how does amyloid *in vivo* get to be deposited at particular cell or tissue sites and does this tell us anything about current concepts that are based on *in vitro* studies?

## Pathogenesis of amyloid deposition *in vivo*

During the 1890s it was noticed that immunization of horses for the production of diphtheria antisera led to systemic amyloidosis. Rabbits and mice immunized with foreign antigens also developed systemic amyloid deposition. These observations raised the possibility that a disturbance of the immune system played a role in amyloidogenesis. This concept was consistent with clinical and histological observations though no clearly defined mechanism was invoked. In the early 1970s, when this vague immunological idea was still prevalent, experiments were performed to determine whether “immunological memory” (in the sense that the immune system “remembers” previous exposure to defined antigens and reacts much more rapidly on second exposure to the antigen than following the first) played a role in amyloidogenesis (Axelrad et al., 1975, 1982; Axelrad and Kisilevsky, 1980). The results of these investigations inadvertently led to the recognition of a biological property called “amyloid enhancing factor” (AEF) which, on the basis of kinetic data, appeared to function *in vivo* as a “seed” for fibrillization (Kisilevsky and Boudreau, 1983). Furthermore, in the presence of AEF any acute inflammatory stimulus, immunogenic or not, could very rapidly trigger amyloid deposition of the AA type (Axelrad et al., 1975, 1982; Axelrad and Kisilevsky, 1980). This observation, among others to be considered below, began to question whether an immune process was at the basis of amyloid deposition generally.

## Amyloid composition and structure

It is generally accepted that the first description of organ involvement by what we now consider to be amyloid was made by Nicolaus Fontanus in 1639. Little additional understanding occurred for another 150–200 years. The possibility that lipid-like material was the basic nature of amyloid was implied by the descriptive term “lardaceous” used by Portal, Merat, and Rokitansky in reports of the period 1789–1842 (cf Kyle, 2001). “Amyloid” meaning starch-like (from the Greek “amylon” for starch) was coined by Schleiden in 1838 (cf Kyle, 2001) for botanical purposes and was applied (Virchow, 1854) to organ amyloid based on its positive reaction with iodine in an acidic environment, the chemical reaction being used to identify starch or cellulose. Virchow's interpretation of the positive result was that amyloid was starch-like in nature, which implied that it was a carbohydrate. His conclusion was not completely accurate. Nonetheless, the name persists as does the concept that a carbohydrate is part of amyloid deposits *in vivo* (discussed below).

Initial histological observations (1859–1920) based on dye interactions with tissue sections suggested that the amyloid deposits were “albuminoid” in nature (Friedrich and Kekule, 1859), namely protein, and possessed no particular organization (cf Kyle, 2001). The development of Congo red in 1883 for use in the textile industry and subsequent use for the staining of tissue sections for microscopy was instrumental in changing this view (Bennhold, 1922). The red/green birefringence observed in Congo red stained tissue sections when viewed in polarized light indicated a very well ordered repetitive super-structure which conferred the characteristic optical properties to amyloid upon its binding of Congo red (Howie et al., 2008; Howie and Brewer, 2009). However, the nature of this structure remained elusive until the 1950s.

The advent of the electron microscope and its application to amyloid tissue deposits (1950s) revealed the fibrillar nature of amyloid (Cohen and Calkins, 1959), changed our concept of its structure and provided a specific direction for amyloid research to follow. The challenge was to isolate these fibrils and then determine the protein responsible for its structure (singular, since at that time amyloid was thought to be the same regardless of its tissue of origin). In the late 1960s groups in Israel (Pras et al., 1968; Franklin and Pras, 1969), and Boston (Cohen and Calkins, 1964), developed techniques to liberate fibrils from tissues containing amyloid and it was assumed that the structure of such liberated fibrils (*ex vivo* fibrils) was identical to those found *in situ*. This may or may not be true and will be addressed in greater detail below as it bears on several additional concepts and conclusions that are driving current research. Regardless, this technical achievement led to an explosion of information concerning the structure of *ex vivo* and *in vitro* fibrils, their composition, identification of the various amyloidogenic peptides and their protein precursors, and characterization of structural modifications and intermediates that occur during the *in vitro* conversion of the globular precursor into fibrils. It provided the impetus to:

1. determine where and under what conditions biosynthesis of each of the amyloidogenic proteins took place,
2. conduct genetic analyses and distribution of specific forms of amyloidosis in human populations,
3. identify particular mutations that enhance the amyloidogenic potential of the protein concerned, and,
4. develop techniques for the *in vitro* study of fibrillogenesis to elucidate the kinetics and thermodynamics of protein fibril assembly and the fine structure of such fibrils.

In 1970 the first amyloid protein was isolated from natural deposits occurring in a patient affected by multiple myeloma and amyloidosis. This amyloid protein was composed mainly of a fragment of a monoclonal light chain whose primary structure was identical to the variable region of the monoclonal light chains isolated from the patient's urine (Glenner et al., 1970, 1971a,c). At that time amyloid was still thought to be the same regardless of its clinical setting or tissue of origin, and the observation that an immunoglobulin could form amyloid was consistent with the then prevailing view that amyloid was a product of a derangement in immune function. Additional evidence supporting this idea came from the clinical settings in which amyloid was frequently seen such as persistent infections (e.g., tuberculosis, osteomyelitis), auto-immune diseases (e.g., rheumatoid arthritis or ankylosing spondylitis) and malignancies of the immune system (e.g., multiple myeloma and B-cell dyscrasias). Moreover, the cell types seen in the tissues affected in such diseases were lymphocytes, macrophages, and plasma cells, cells of the immune system. Not surprisingly between the 1890s and 1970 the primary concept concerning the pathogenesis of amyloid was a poorly defined disturbance of immune function in which proteolytic fragmentation of the immunoglobulin light-chain led to the deposition of these fragments as amyloid. This conclusion was supported by *in vitro* studies with Bence-Jones protein (the monoclonal light chain present in urine) which when treated with trypsin generated fibrils that appeared identical to those extracted from tissue (Glenner et al., 1971a; Glenner, 1972). Analysis of these *ex vivo* fibrils, and those created *in vitro*, by X-ray diffraction and subsequently infra-red spectroscopy revealed an underlying crossed beta-sheet organization (Glenner et al., 1971b; Harada et al., 1971).

Thus, by the early 1970s, the accumulated data laid the foundation for most of the concepts driving current investigations. A more detailed definition of amyloid was framed: when examined *in situ* amyloid appeared amorphous with routine stains and light microscopy; amyloid stained with Congo red when viewed in polarized light exhibited red/green birefringence; ultrastructurally amyloid was composed of fibrils; and fibrils extracted from tissue as well as fibrils generated *in vitro* possessed a characteristic X-ray diffraction pattern. Though pathologists in the 1930's observed subtle staining differences with Congo red between primary (now AL) and secondary (now AA) amyloid which suggested an underlying difference in chemical composition, in the late 1960s to early 1970s, amyloid was still considered to be a product of a single protein, likely an immunoglobulin light-chain, and proteolysis was believed to be a critical step in its conversion to fibrils. Furthermore, the fibrils seen *ex vivo* and those generated *in vitro* were thought to be identical to those seen *in situ*.

Substantial progress has been made regarding protein fibril structure since the early 1970's. A large literature is now available which describes the kinetics and thermodynamics of amyloid-like fibril assembly *in vitro*, and under appropriate *in vitro* conditions virtually any protein has the potential to change conformation and acquire substantial beta-sheet structure (cf Buell et al., 2014). In the last 4–5 years the application of protein magic angle spin solid state NMR to the structural characterization of *ex vivo* amyloid fibrils from different proteins is providing fundamental atomic details on the structure of amyloidogenic monomers and the type of intermolecular interactions that exist between these monomers (Petkova et al., 2006; Wasmer et al., 2008; Barbet-Massin et al., 2010; Debelouchina et al., 2010, 2013; Comellas et al., 2011; Hellmus et al., 2011). Through solid state NMR in combination with high resolution cryo-electron microscopy and atomic force microscopy the general 3D structure of *ex vivo* amyloid fibrils is emerging as well as the specific structural differences contributed by the different types of amyloidogenic proteins. Recent work (Lu et al., 2013) on the structure of Aβ amyloid fibrils seeded on Aβ amyloid plaques in brain homogenates attempted to exploit the potency of this technology in revealing subtle but important differences between fibrils obtained *in vitro* and fibrils grown on a substrate of natural Aβ amyloid plaques, or possibly the extracellular matrix (stroma) components within such plaques (Snow et al., 1988, 1989; Narindrasorasak et al., 1991). Though differences in Aβ fibril structure were observed in the presence and absence of Aβ plaques in the homogenates the results also proved different from *in vitro* Aβ fibril assemblies as seen with cryo-electron microscopy (Fandrich et al., 2011). It is still not clear which of these results reflect the *in situ* situation. The presence of unexpected twists in particular strands, or novel orientation of side chains of specific amino acid residues, as a result of the influence of the plaque or its stroma, may be very relevant in dictating the physical properties of fibrils *in situ.* They may also influence the kinetics of fibrils growth and generate specific conformations suitable for the binding of various ligands. These data raise the distinct possibility that *in vivo* components of tissue stroma have a role in the structure and potentially the anatomic site of the deposition process itself. This idea is consistent with the known presence of such components, e.g., serum amyloid P (SAP), apolipoprotein E (apoE), glycosaminoglycans (GAGs) such as heparan sulfate (HS), HS proteoglycan (HSPG), laminin and particular forms of collagen, in amyloid fibrils *in situ* (Lyon et al., 1991). Experimental studies examining fibrillogenesis of different amyloidogenic proteins on different tissue, or synthetic, matrices will probably become a ripe area for investigation and may succeed in enlightening us about the possible differences and similarities between amyloid-like fibrils *in vitro* and *in situ* amyloid fibrils. Initial explorations in this direction have already begun (Mazza et al., 2016).

Notwithstanding the substantial advances made understanding protein fibrillogenesis and fibril structure *in vitro*, progress regarding these processes *in situ/in vivo* has not proceeded apace. Currently, structural comparisons of *ex vivo/in vitro* fibrils with those observed *in situ* rest on relatively few studies. One similarity is their common Congo red and thioflavin T staining properties. There are but two infra-red studies of *in situ* amyloid, one examining procalcitonin and the other Aβ amyloids *in situ* (O'Leary and Levin, 1985; Choo et al., 1996). There is but a single X-ray microprobe study of Aβ *in situ* (Briki et al., 2011), a single cryo-fixation, freeze substitution and standard EM study of AA amyloid fibrils *in situ* (Inoue and Kisilevsky, 1999; Inoue et al., 2002), and one study of transthyretin amyloid fibrils extracted from the vitreous of the eye from a patient with a familial form of ATTR (Serpell et al., 1995). Whether the large body of information on *in vitro/ex vivo* fibrils and the limited structural information on *in situ* fibrils is sufficient to establish equivalence between the two sources of fibrils has yet to be settled.

The understanding of fibril composition and structure achieved from 1970 onwards rendered incorrect some pre-existing concepts concerning the make-up of amyloid and its pathogenesis and required significant amendments. In 1970–1972 data emerged from several groups that at least one amyloid protein was not related to immunoglobulins (Benditt et al., 1971; Benditt and Eriksen, 1971, 1972; Ein et al., 1972; Hermodson et al., 1972). In 1970–1980, in addition to the AEF studies which questioned the role of immune mechanisms in AA amyloidogenesis, it became apparent that several different proteins could be responsible for amyloid deposits *in vivo*. During this period at least a dozen such proteins were identified and the number has now climbed to over 30 (Westermark et al., 2007). Each amyloid protein was shown to be associated with a distinct clinical disorder or pathologic process. The older idea that amyloid was always due to the same protein, regardless of tissue source, or clinical disorder, had to be discarded. Furthermore, many of the identified proteins had little, if anything, to do with immune function and the concept that an undefined immunological disturbance was at the basis of all amyloids has also been discarded. Moreover, not all of the identified amyloidogenic proteins appeared to require proteolysis for fibril assembly. There are multiple examples in which the full length unmodified protein is present in the natural fibrils which include SAA in ducks (Ericsson et al., 1987), a mutant variant of lysozyme (Pepys et al., 1993), and the natural variant of β2M (Valleix et al., 2012). Nor was it clear whether in some cases proteolytic cleavage was a pre- or post-fibrillogenic *in vivo* event. Since many of the tissue amyloid deposits had existed *in vivo* for months or years prior to their isolation and examination it remained possible that *in vivo* proteolytic attack occurring after deposition could generate a family of related peptides that could be purified from the isolated fibrils (Kisilevsky et al., 1994; Röcken et al., 2000; Enqvist et al., 2009). It is also possible that truncated species of a protein precursor, chemically identical to those found in natural fibrils, possesses strong amyloidogenic propensity (Esposito et al., 2000; Mangione et al., 2014), and can generate a fibril nucleus able to catalyze the aggregation of the less amyloidogenic full length protein.

Despite the wealth of basic knowledge on the biophysical basis of fibrillogenesis of native proteins *in vitro* the reasons why relatively few of the 20,000–25,000 proteins encoded by the genome are associated with amyloid deposition in humans and the mechanisms that drive the tissue selectivity of systemic amyloid deposition remains totally unexplained. Nevertheless, the acquired data have raised many questions related to amyloidogenesis, among which are:

1. Is the cross beta-sheet structure of a fibrillar protein aggregate the lowest free energy state of that polypeptide, and is the main driving force for the conformational transition of the amyloidogenic protein *in vivo* the energy landscape in which it exists?
2. Are the *in vitro* fibrillogenic conditions ones that might be seen *in vivo*, and if so where do these occur anatomically?
3. Are the amyloid-like fibrils generated *in vitro* from a single protein really identical to, or only similar to, amyloid fibrils seen *in vivo*?
4. What role do co-precipitating components (e.g., SAP, GAGs, laminin, and type IV collagen of the extracellular matrix) known to be part of *in vivo* amyloid deposits play in amyloidogenesis (Kisilevsky, 2000)? Are they simply “associated” factors, or “critical” components, as suggested by inhibition of *in vivo* fibrillogenesis when one alters the availability of SAP (Pepys et al., 2002; Inoue et al., 2005; Bodin et al., 2010), or the state and/or quantity of HS (Kisilevsky et al., 2007).
5. Has our current focus primarily on proteins identified in amyloid-like fibrils inadvertently altered our definition of amyloid so that it has lost its previous clinical and pathological context? Has amyloid's original definition therefore become distorted to a “protein only” entity and problem? Such a perspective would distract/deflect us from understanding the role of additional components in amyloid structure, the more complex details of fibrillogenesis *in vivo* involving these additional components and potentially prevent us from considering broader therapeutic possibilities derived from an understanding of these complexities.
6. What roles do natural inhibitors of protein mis-folding, and stabilizers of protein conformation, (the “chaperones”) play in amyloidogenesis *in vivo*?
7. How does a critical concentration of an amyloidogenic protein arise at anatomic sites remote from its site of biosynthesis?
8. How do amyloid deposits influence cell and tissue function and viability?

**Figure 3:**
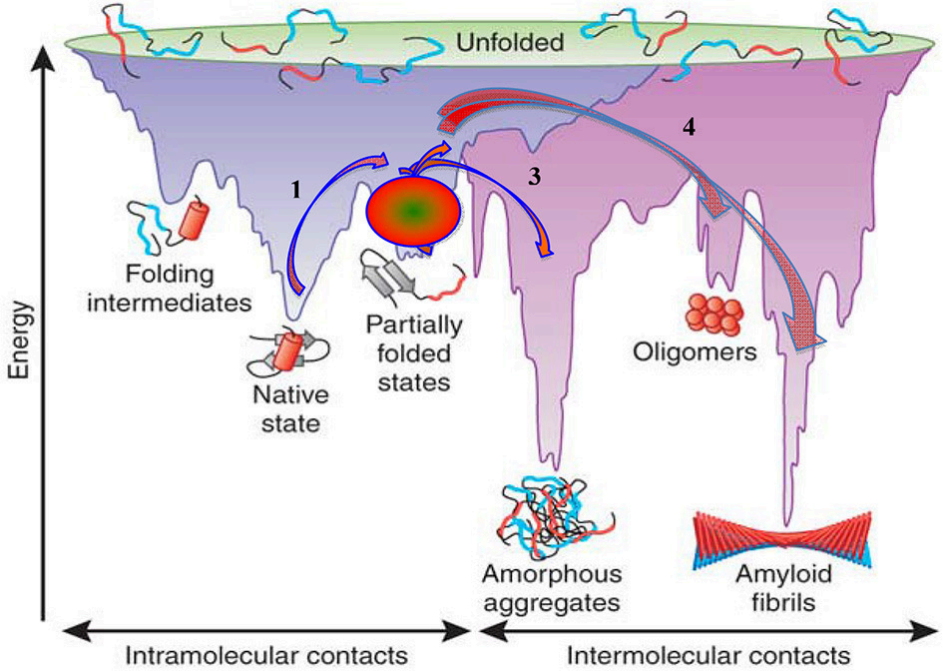
**Energy landscape scheme of protein folding and aggregation**. Reproduced, with permission, from Hartl and Hayer-Hartl (2009).

The basic concepts concerning protein folding and misfolding and their possible relationship to protein fibril assembly and amyloid formation are schematically presented in Figure 3, which is reproduced from Hartl and Hayer-Hartl (2009). In this scheme fibril formation is driven thermodynamically by the very low free energy of the fibrillar state. This deep free energy minimum is derived from the extensive formation of intermolecular hydrogen bonds, hydrophobic interactions and water exclusion from the inner core of the fibril. In the case of systemic amyloidoses the pathway of amyloidogenesis begins with native globular proteins that circulate freely in plasma, diffuse into the interstitial space but ordinarily possess a “local” free energy minimum that, in effect, prevents them from cascading further down the free energy scale unless they are perturbed to overcome this energy barrier. *In vitro* this barrier can be “softened” by altering the conditions in which the globular protein exits, such as increasing the temperature, reducing the pH, introducing organic solvents, changing the sequence of the normal protein, or removing an associated protein (Hatters et al., 2001; Rekas et al., 2004; Raman et al., 2005; Mangione et al., 2013). The sources providing sufficient energy to overcome the barrier *in vivo* are still largely undetermined and some proteins intrinsically amyloidogenic in *in vitro* experiments never overcome the barrier *in vivo*. Wild-type lysozyme is an example of a potentially amyloidogenic protein that never appears to enter the amyloid pathway. However, mutations may alter the minimum free energy of the native, and or, the intermediate states, effectively altering the free energy landscape making it possible (step 1, Figure 3) to proceed to a partially unfolded intermediate state. This intermediate and metastable state (step 2, Figure 3) appears to be a key step in any further transformation and a crucial entity in amyloid conversion. The intermediate state is most likely a population of heterogeneous conformers whose structural characterization is extremely difficult due to their rapid inter-conversions. The formation of an intermediate state *in vivo* has not yet been demonstrated but all *in vitro* evidence would indicate this also occurs in living systems.

A necessary property of any amyloidogenic globular protein appears to be its folding instability and almost all the methods of *in vitro* fibrillogenesis are based on conditions inducing a partial protein unfolding (Bellotti and Chiti, 2008). Several groups have investigated the fibrillogenesis of β2M which provides a good example of the evolution of concepts and methods related to the formation of amyloid-like fibrils *in vitro* (Stoppini and Bellotti, 2015). This progressed through the use of full length β2M and fibrillogenic seeding at low pH, alterations in salt composition and concentration, use of truncated, or partially unfolded, forms of β2M, introduction of GAGs at more neutral pH, use of phosphate buffers and the use of a natural amyloidogenic variant (Naiki et al., 1997; Esposito et al., 2000; McParland et al., 2000; Chiti et al., 2001; Yamamoto et al., 2004a,b; Jahn et al., 2006; Relini et al., 2008; Eichner and Radford, 2011; Mangione et al., 2013).

Recent additional work on the first natural amyloidogenic variant of β2M has highlighted the role of shear forces generated by the flow of physiologic fluids and the exposure of this variant to hydrophobic surfaces (Valleix et al., 2012; Mangione et al., 2013). In particular in this variant the replacement of Asp76 by Asn destabilizes the folded state by 2.8 kcal/mol. Nevertheless, in the cell, this variant is fully folded and functions properly through its stabilizing interaction with the Class I major histocompatibilty complex (Halabelian et al., 2014). A further property of this mutant form of β2M is that the single amino acid substitution drives the anatomic site of amyloid deposition. In fact the wild-type β2M is found primarily in large joints, bones and ligaments but the mutant form is deposited systemically.

## Amyloid cell and tissue targeting *in vivo*

*In vivo* deposition of amyloid may occur at, or close to, the biosynthetic site of its amyloidogenic precursor, or at a site remote from its site of synthesis. When at or near the site of biosynthesis, fibrillogenesis may occur within the cell or close to it extracellularly. Examples of the former are fibrils found within light-chain synthesizing plasma cells in multiple myeloma (Ishihara et al., 1991), phosphorylated tau fibrils found in neurons containing neurofibrillary tangles (Ruben et al., 1992; Snow et al., 1992), islet amyloid polypeptide (IAPP) in β-cells of the islets of Langerhans (de Koning et al., 1994b; Westermark et al., 2011) and amyloid-like fibrils seen in various species of yeast (Wickner et al., 2013). In each of these situations one can imagine an intracellular synthesized protein experiencing conditions (mutations, temperature, pH, shear forces, protein concentration, or lack of proper chaperones) that alter the stability of the folded protein creating a micro-environment conducive to the generation of conformational intermediates, oligomer formation, and finally fibril formation as one sees *in vitro.*

In addition to being a protein that undergoes intracellular fibrillogenesis IAPP is also an example of extracellular amyloid deposited close to its site of synthesis. It is believed that Aβ deposits in the central nervous system in Alzheimer's disease may represent another such example. These proteins are synthesized by local cells and after their export may find, in the immediate vicinity of this cell, conditions conducive to the generation of conformational intermediates, oligomer formation and then fibril deposition. However, IAPP is instructive for another reason. Its fibrils are found throughout the islets though not beyond its confines (de Koning et al., 1994a; Ma et al., 2000). If this protein, or its initial conformational intermediates, can diffuse beyond its cell of origin why does the amyloid end abruptly at the endocrine:exocrine interface and not also involve the immediately adjacent exocrine portions of the pancreas? What are the factors that restrict its deposition to the islets? The converse to this question is, what keeps some amyloidogenic proteins from being deposited as amyloid at/near their site of synthesis, but allows them to be deposited at other anatomic sites.

There are many forms of amyloid that fall into the class of “extracellular amyloid deposition remote from the site of synthesis of their protein precursors.” In fact most clinically relevant forms of systemic amyloidosis belong in this category and include AA amyloid (a consequence of persistent acute inflammation), AL amyloid (a consequence of myeloma or B-cells dyscrasias), ATTR (transthyretin amyloid, many forms of which are a consequence of single amino acid substitutions), Aβ2M (a consequence of long term hemodialysis), and several additional forms of amyloid such as those derived from fibrinogen (Asl et al., 1997) or apoA-I (Amarzguioui et al., 1998; Benson, 2001). This list is not complete. Among the many examples that can be discussed only AA, ATTR, and Aβ2M will be considered. Together they identify questions that need to be addressed if we are to understand, sift, and apply the information from studies of *in vitro* fibril formation to the *in vivo* setting.

The precursor to AA amyloid, SAA, is synthesized primarily in the liver and during an inflammatory reaction its plasma concentration may increase 1000 fold (McAdam et al., 1978). However, the deposition of SAA as AA amyloid occurs first in the spleen specifically the perifollicular zone within this organ as seen in mice and mink (Husby et al., 1975; Snow and Kisilevsky, 1985), and extensive follicular splenic deposition in humans (Buck and Koss, 1991; the “sago spleen” in Fontanus' original description of 1639). Why is this so? The follicle is the filtering zone within the spleen and one may argue that high plasma concentrations of SAA may lead to the formation of conformational intermediates and oligomers which are cleared by the spleen. If it is true, as shown *in vitro*, that conformational intermediates and oligomer formation is a common pathway from amyloid precursors to amyloid deposits then other amyloid precursors that exist in plasma should also follow this anatomic deposition pattern. This however is not the case. This follicular amyloid distribution is peculiar to AA amyloid. TTR, the precursor to ATTR, is also made primarily in the liver but its varying anatomic distribution as amyloid appears to be related to particular mutations. Wild-type TTR is seen mainly in the heart and in older individuals (Benson, 2012), the peripheral nervous system is the preference of one mutation (Benson and Uemichi, 1996), in another mutation deposition is almost exclusively in the heart (Buxbaum et al., 2010) and in yet another it is apparently in the leptomeninges (Benson, 1996; Nakagawa et al., 2008). The spleen is rarely, if ever, involved in ATTR regardless of the presence or absence of mutations. Similarly, fibrinogen and apoA-I are synthesized in the liver but are deposited in extra-hepatic sites. It appears therefore that if protein concentration, conformational instability, and oligomers play the role proposed *in vitro* it is more likely that the unstable forms and early aggregates are generated not close to the cell which synthesizes the precursor or in plasma but more likely in the micro-locale where these proteins are finally deposited. We know very little about potential micro-environmental factors (ligands, temperature, pH, shear forces, protein concentration, or removal of chaperones), how they are generated, how they determine which organ is targeted by the different amyloidogenic proteins, and in particular why single amino acid substitutions in a specific amyloidogenic protein (e.g., TTR, apoA-I, or β2M) changes the organs involved.

## Potential factors influencing tissue targeting

The fact that single amino acid substitutions can change the tissue site of amyloid deposition suggests that some of the information determining this deposition resides in the structural and functional features of the amyloidogenic protein itself. But with what does the amyloidogenic protein interact and what is the effect of this environment? Though possible, it is unlikely that the ionic composition of interstitial fluid in tissue stroma varies significantly in different organs, or from one locale to another within the same organ. This suggests that attention should focus on larger molecular entities with which the amyloidogenic proteins may interact. Several possibilities come to mind.

1. **Protein:protein interactions** in the extracellular matrix are extremely varied and span a large range of affinities, from the low affinity of chaperones proteins to the high affinity of specific receptors. An example of the latter is the high affinity binding of SAA, the AA precursor, to laminin, a stromal protein. SAA has been shown to compete with entactin (a normal laminin ligand necessary for the building of basement membrane structures) for the laminin:entactin binding site (Ancsin and Kisilevsky, 1997, 1999). The interaction with the hydrophobic surfaces of fibrous proteins, constitutive components of the extracellular matrix, is also extremely important although the interaction is not specific for the amyloidogenic proteins. However, the effect of the interaction of hydrophobic surfaces and globular amyloidogenic proteins may be sufficiently specific that once such contact is made the amyloidogenic protein can unfold locally and its propensity to self-aggregate enhanced (Husby et al., 1994; Relini et al., 2006, 2008).
2. **Protein:proteoglycan binding**, particularly HSPG and its HS side chains. There is a substantial literature implicating HS in amyloidogenesis *in vivo* (Kisilevsky et al., 2007). Though HS has a common repeating disaccharide backbone that is the same from tissue to tissue and cell to cell, there is extensive variation in its pattern of sulfation and epimerization to account for different specificities between different tissues and for different specificities within the same tissue. Furthermore, these HS structural variations change with age and physiological states (Feyzi et al., 1998; Parish, 2006) and may explain why amyloids are more common later in life. Shear forces have been implicated in the clustering of HS (Zeng et al., 2013) and both of these factors have been shown to have an effect on protein fibrillization. Heparan sulfate has also been shown to play a role in dissociating SAA from HDL, its normal carrier in plasma (Noborn et al., 2012), and such a separation of SAA from HDL may alter SAA's conformational stability. Precisely how proteins bind/interact with HS, where their complimentary binding faces exist and how the specificities of protein binding are determined is a subject that cuts across many areas of research (Kisilevsky and Ancsin, 2010). Nevertheless, this binding has been used successfully as a therapeutic target in treating AA amyloidosis in animals and humans (Kisilevsky et al., 1995; Dember et al., 2007).

## Cell and tissue injury caused by amyloid deposits

At least two pathological mechanisms are apparently involved in amyloid causing cell and tissue injury. There may be others to be discovered in the future. Historically, the first mechanism is based on the gross and microscopic appearance of amyloid as seen at the organ and tissue levels (Kisilevsky, 2007). Organs infiltrated with large quantities of amyloid, regardless of type, become rigid and this rigidity may affect their function (e.g., the heart). At a histological level amyloid is deposited between blood vessels and parenchymal cells using the stromal architecture of the extracellular matrix (Kisilevsky, 2007). This is believed to impair the transfer of nutrients to, and of metabolic and functional products from, parenchymal cells affecting their physiological function. Histological observations also suggest that amyloid *in situ* surrounds parenchymal cells constricting the space about these cells (Kisilevsky, 2007). Such processes altering cell viability and function were suggested decades before the fibrillar nature of amyloid was understood. These older perspectives are no longer fashionable but there is little experimental evidence that rules them out. Additional proposals have arisen in the last 20 years based on cell/tissue culture data. Attempts to determine if large amyloid deposits, as seen histologically, had adverse properties *vis a vis* cell viability and function in culture proved disappointing. However, oligomeric units (small aggregates, not necessarily fibrillar) of amyloidogenic proteins did influence cell viability (Haass and Steiner, 2001; Glabe, 2006). It was therefore proposed that small fibrils/oligomers, rather than large amyloid aggregates formed *in vivo* were responsible for cell toxicity and altered cell function seen *in vivo*. This conclusion needs to be tempered, and the dismissal of the *in vivo* cell and tissue effects of large amyloid deposits is premature because the anatomic relationship of large aggregates to cells in culture is not analogous to amyloid seen in the extracellular matrix between cells, and between cells and capillaries *in vivo*. Nevertheless, there is ample evidence that monomers and oligomers do have an effect on cell viability in culture and *in vivo*. An interesting example of the former is impaired cardiac function in AL amyloid and its positive correlation with the plasma level of free amyloidogenic light chains rather than the quantity of amyloid deposited in the heart (Palladini et al., 2006). However, in these patients to fully express their toxicity the soluble prefibrillar species requires the presence of amyloid deposits in the tissues.

Additional studies have revealed that amyloidogenic monomers can form beta barrels and they, as well as oligomers, can insert themselves into cell membranes (plasma membranes and intra-cellularly) altering cell permeability, or creating membrane pores and impairing mitochondrial and endoplasmic reticulum function with devastating consequences (Haass and Steiner, 2001; Glabe, 2006). Monomers, oligomers as well as large amyloid deposits may be operative *in vivo* but only the first two can be demonstrated in culture. Furthermore, *in vivo* their effects may be additive or synergistic and their adverse effects fully expressed when both fibrils and precursor are abundantly present. Additionally, amyloid fibrils in the extracellular matrix may enhance the mis-folding and aggregation of adjacent corresponding precursor globular proteins (Bellotti and Chiti, 2008). To study such questions it may be possible to generate scaffolds that mimic the extracellular matrix and use them as tools to study the interplay between matrix, amyloid precursors, and amyloid fibrils.

## Summary

A consideration of the general determinants that govern which tissues are targeted by which amyloidogenic proteins is an aspect that at present is not being, or cannot be, addressed/assessed *in vitro*. The generation of critical protein concentrations and the behavior of conformational intermediates and oligomers *in vitro* do not necessarily indicate how and where these are formed *in vivo*. They are not likely to be formed adjacent to the cells synthesizing the protein nor in plasma as, being particulate, they would likely be cleared by the reticulo-endothelial system and the different amyloids would have very similar tissue distributions. This suggests that conformational intermediates and oligomers are generated close to the site of amyloid deposition, and are subject to the same factors that determine the specific tissue distribution of amyloids.

## Author contributions

RK and VB provided the majority of the thought concerning the concepts considered and did the majority of the writing. SR assisted in the organization of the paper tracking the references and organizing the bibliography.

## Funding

Please note that the manuscript is not a typical review, but a perspective and review of the concepts that have driven amyloid research over the last 350 years. RK and VB have been conducting basic and applied research in this area for a combined total of more than 75 years and have been funded by dozens of agencies during this time. We sincerely thank all these agencies, which are each listed in the respective publications which they supported, and, for their very generous support over the period 1970—present. We could not have achieved our contributions and nor developed our perspectives in this research area in the absence of such wide support.

### Conflict of interest statement

RK has patent and royalty interests in compounds that interfere with HS:amyloidogenic protein interactions as potential anti-amyloid therapies, and patent interests in SAA related peptides that have anti-atherogenic properties. The reviewer LB and handling Editor declared their shared affiliation, and the handling Editor states that the process nevertheless met the standards of a fair and objective review.

## Abbreviations

AA: amyloid A
Aβ: amyloid-beta
Aβ2M: beta-2-microglobulin amyloid
AEF: amyloid enhancing factor
AIAPP: islet amyloid polypeptide amyloid
AL: immunoglobulin light-chain amyloid
apoAI: apolipoprotein AI
apoE: apolipoprotein E
ATTR: transthyretin amyloid
*β*2M: beta-2-microglobulin
GAG: glycosaminoglycan
HDL: high density lipoprotein
HS: heparan sulfate
HSPG: heparan sulfate proteoglycan
IAPP: islet amyloid polypeptide
NMR: nuclear magnetic resonance
SAA: serum amyloid A
SAP: serum amyloid P
TTR: transthyretin.
